# Continuity within a community integrated palliative care model and the influence of remote and digital approaches to care: a qualitative interview study

**DOI:** 10.3399/BJGPO.2024.0126

**Published:** 2025-02-26

**Authors:** Emma Ladds, Malaika Ivey, Katrina Gadsby, Elin Preest, Ffion Samuels, Victoria Bradley

**Affiliations:** 1 Nuffield Department of Primary Care Health Sciences, University of Oxford, Oxford, UK; 2 University of Oxford Clinical Medical School, John Radcliffe Hospital, Oxford, UK; 3 Oxford University Hospitals NHS Foundation Trust, John Radcliffe Hospital, Oxford, UK

**Keywords:** Terminal illness and palliative care, integrated care, remote consultation, digital health, qualitative research

## Abstract

**Background:**

Demand for palliative care is rising. Recent UK policy approaches promote integrated care models — collaborations between GPs and multidisciplinary specialists — and remote and digital practices. The extent to which different forms of continuity are supported within this evolving context is currently unclear.

**Aim:**

To explore the experience of continuity and impact of remote and digital practices within an integrated palliative care model.

**Design & setting:**

A qualitative interview study of patients and bereaved relatives recruited from a GP practice list and healthcare professionals delivering the integrated palliative care service for that population.

**Method:**

Twenty narrative and semi-structured interviews were conducted with 22 patients, relatives, and professionals between May 2022 and November 2023. They explored how care was delivered or received, focusing on coherency and the use of remote and digital practices. Data were theorised using a novel framework that considered psychodynamic, biomedical, sociotechnical, and sociopolitical domains of continuity.

**Results:**

The need for human care and connection were of primary importance and affected by intersubjective, biomedical, sociotechnical, and sociopolitical factors that influenced continuity of care. Despite the logistical ease of remote and digital practices, professionals had to work harder or around technologies to provide a ‘caring’ service. This was exacerbated by a lack of co-localisation, loss of longitudinal relationships, and reduction in tacit knowledge.

**Conclusion:**

Numerous complex factors and the exacerbating effects of remote and digital practices influence continuity and coherency within an integrated palliative care model.

## How this fits in

The extent to which continuity and a coherent care experience are enabled within an integrated palliative care model is currently unclear. Here we explore intersubjective, biomedical, sociotechnical, and sociopolitical factors that may influence continuity and coherency. We reveal transactional and relational levels of information and knowledge within these that may be incompletely communicated using remote and digital approaches to care, and as a result of alterations to traditional working patterns. We highlight the need for policy makers to consider how best to enable interprofessional information sharing on both levels to ensure the highest coherency within an integrated approach.

## Introduction

The demand for palliative and end-of-life care in the UK is rising, driven by an aging, multimorbid population.^1^ Palliative care relates to the holistic support offered to patients and their families at any stage following diagnosis of incurable illness. Day-to-day care is provided within the community by relatives and paid carers, residential home staff, district nursing teams, GPs and other professionals with the aim of providing comfort and quality of life in accordance with the patient’s wishes. When more expert knowledge is required, specialist multidisciplinary palliative care services can provide support.^2^ A small minority benefit from inpatient care. Some individuals currently receive suboptimal care due to overstretched services^3^ with significant inequalities arising from geographic, socioeconomic,^4^ and digital/technological factors.^5^

High-quality palliative care should enable a coherent, person-centred experience with minimal unplanned hospital admissions or interventions discordant with the patient’s wishes. Such admissions are increasing, causing distress for individuals and expense to the healthcare system.^6^ The proportion of patients experiencing one within the last year of life rose from 21% to 25% between 2009 and 2018 and those with 3 or more in the last 3 months of life increased from 5.6% to 7.1%^7^. The Buckinghamshire, Oxfordshire, Berkshire West (BOB) region (where this study is sited) performed significantly worse than the national average.^8^

Given this context, the 2022 Health and Care Act included a requirement for local Integrated Care Systems (ICSs) to provide and prioritise palliative care services with the support of seven regional Palliative and End-of-Life Care (P&EOLC) strategic clinical networks.^9,10^ However, commissioners frequently have limited knowledge or oversight of generalist providers^11^ and specialist palliative care, hospice, and community services are highly variable.^12^ Moreover, the geographically-distinct NHS, private, and voluntary providers have distinct professional identities, working practices, technological and information infrastructures, and strategic relationships that can hinder implementation.

Given this landscape, effective communication and coordination of care is challenging. Evidence suggests that patients with advanced disease often experience deficiencies in relational, managerial, and informational continuity.^13,14^ Integrated models may provide a compensatory structure, enabling more coherent care.^15^ However, the evidence in practice is limited. Furthermore, digital and remote approaches to care, promoted by recent UK health policies, add further complexity. Examples include asynchronous communications and virtual consultations, digital patient records, and electronic prescribing. Such approaches may distribute care in space and time,^16,17^ induce disembodiment,^18–20^ challenge empathic connection and disrupt therapeutic presence^21,22^ — all particularly salient in terminal conditions.

The challenge of enabling coherency within this context is captured in a recently developed framework of continuity. This considers four domains: the psychodynamic (intersubjective relationship), biomedical (disease/illness episode), sociotechnical (work of care); and sociopolitical (community/wider system).^23^ All must be considered to produce a person-centred, coherent experience. Here we use this understanding to analyse patient, family, and professional experiences of an integrated palliative care model and explore the challenges facing the 2022 Health and Social Care Act.

## Methods

### Study design and data collection

Data were collected as part of a wider project — the Remote-by-Default 2 study —which is described in detail elsewhere.^24^ Twenty interviews were conducted in two phases: May 2022–July 2022 and May 2023– October 2023. Participant demographic information is summarised in Table 1. In the initial phase, MI, a clinical medical student, conducted seven narrative interviews with two patients with terminal diagnoses, five recently bereaved relatives, and two nursing/residential/dementia care home staff (manager and nurse) (some interviews involved more than one participant). Participants were recruited from the lead author’s GP practice. A narrative approach was used with a single opening question, *‘tell me about your experience of palliative care’,* which centred the interview around the individual(s), their story, and the interpretations and meaning they ascribed to it. Interviewees told the story, uninterrupted, in their own words, with the interviewer using conversational prompts (such as ‘what happened next?‘ or ‘how did you feel when that happened?’) to maintain the narrative.^25^ Narrative interviews may be particularly useful for raising sensitive issues and identifying emotional touchpoints in an illness journey,^26^ which may be particularly relevant for those receiving palliative care.

**Table 1.** Summary of participant demographics

	Age band, years	Gender
**Phase 1**		
Patient	55-60	Male
Patient	75-80	Male
Bereaved relative	60-65	Female
Bereaved relative	80-85	Female
Bereaved relative	50-55	Female
Bereaved relative	55-60	Female
Bereaved relative	25-30	Male
Care Home Manager	55-60	Female
Care Home Nurse	50-55	Male
**Phase 2**		
GP	55-60	Male
GP	55-60	Male
Palliative Care Consultant	55-60	Female
Palliative Care Consultant	50-55	Female
Palliative Care Consultant	40-45	Female
Palliative Care Specialty Trainee	30-35	Female
Palliative Care Nurse	45-50	Female
District Nurse	55-60	Female
District Nurse	55-60	Female
District Nurse	25-30	Female
District Nurse	30-35	Female
District Nurse	50-55	Female
Community Matron	55-60	Female

In the second phase, purposive sampling identified multidisciplinary professionals from a range of community and hospital teams providing palliative care to patients registered at the lead author’s GP practice; that is, to the participants who had taken part in the first phase. The teams operate at different geographical scales, thus while the initial participants were limited to a single GP practice list, the healthcare professionals were drawn from GPs in that practice, a district nursing team supporting the whole of West Oxfordshire, and a specialist palliative care team operating across the whole of Oxfordshire. Thus experiences were not limited to a single GP practice. EP and FS, clinical medical students, conducted 13 semi-structured interviews with three palliative care consultants, one palliative care specialty trainee doctor, two GPs, one palliative care community nurse, five district nurses, and one community matron. Individuals were asked to describe their experiences of providing care with the interview schedule designed to explore themes identified as meaningful within the initial patient–carer interviews. These included the distribution and coordination of roles, use of remote and digital approaches, and multiprofessional communication. However, conversational prompts also elicited details beyond these areas, enabling individuals to share broader experiences and examples. Interviews were flexible in length, and were conducted and audiorecorded remotely over Microsoft Teams or in person depending on participant preference.

### Data management and analysis

Recordings were transcribed immediately and verbatim by MI, FS, or EP and subsequently deleted. Transcripts were stored on a secure shared University of Oxford online drive and were pseudoanonymised prior to analysis. NVivo (version 12) was used to identify broad coding categories but the overall analytical process was constructive, bringing key themes together. Modelled on the hermeneutic cycle, this involved producing a preliminary synthesis after the initial interview phase then adding richness as this was reviewed and amended with data from subsequent interviews.^27^ This allowed for a focus on the ‘whole’ reality, rather than a representation of partial elements, situated in the everday reality of an individual’s lived experience. Here we present the findings from the professionals involved in delivering integrated palliative care.

### Theoretical approach

Data from the semi-structured professional interviews were analysed using a novel framework of continuity that captures four domains of care: psychodynamic (intersubjective relationship), biomedical (disease/illness episode), sociotechnical (work of care); and sociopolitical (community/wider system).^23^ Within these, the aims of medicine and values of practitioners were explored using Held’s *Ethics of Care*,^28^ MacIntyre’s concept of ‘internal goods’,^29^ and Starfield’s core elements of primary care.^30^ These can be influenced by aspects of remote and digital practices and changing working patterns. Giddens’ theory of distanciation,^16^ Stones’ work on disembedding,^17^ and Dakin’s theories of facsimilisation and responsibilisation^20^ were used to consider the impact of distributing activities across space, time, and virtual reality. We considered how these influenced interprofessional working and relationships,^31,32^ the therapeutic alliance between patient and doctor,^33^ and the holistic generation of working knowledge.^34^

## Results

### Overview of dataset

Fifteen participants were interviewed during phase two of the study. This produced around 100 pages of transcribed data.

### The aim of palliative care

‘Caring’ and ‘comforting’ were seen as fundamental elements of palliative care alongside alleviation or ‘curing’ of physical symptoms. Several participants emphasised the importance of a holistic approach that considered four domains, that is, with spiritual and biopsychosocial factors. Providing the biomedical knowledge and understanding to facilitate an honest contemplation of death alongside the intersubjective connection, space and time for both reflection and meaningful conversations were seen as key components to this.

Such an emphasis resonates with wider debates about the aim(s) of medicine. Specialisation and technological innovation have increased the possibilities to do more to extend life, but potentially also prolong pain, suffering, and disability. Heath and Montori have written movingly about the *‘crisis of care’* in our modern system^35^ that results from a failure to acknowledge that ‘*the map of biomedical science only roughly matches the territory of human suffering’.* ^36^ Nowhere is this more evident than towards the end of life. In contrast, Held’s *Ethics of Care* emphasises the importance of attending to the needs of others to truly *care* for them, recognising the role of emotions and relational connection.^28^ This aligns with the ‘internal goods’ envisaged by MacIntyre that enable a virtuous practice of medicine — essential for the ‘flourishing’ of both patient and practitioner.^29^

### Psychodynamic continuity: the intersubjective relationship(s)

For many participants, this combination of intersubjective and biomedical care was vital. Several professionals emphasised the importance of acknowledging the uncertainty, fear, and isolation faced by patients and families, which could be mitigated through information. GPs described being:

‘... *clear to the supporting family and carers about “what if“ scenarios and how they can best seek help during such episodes and then explaining to carers and relatives what happens when someone dies and what they might observe.’* (JC, male GP in his 50s)

The community matron described how:

*‘... they* [patients and carers] *can just feel really out there and on their own and not knowing where to go.’* (CM).

Such intersubjective care was felt to be less successfully enacted through remote and digital approaches, although such contacts were more acceptable within an existing relationship. Several clinicians emphasised how an in-person meeting was *necessary* for a proper assessment and full understanding, helping to build relational knowledge. One of the GPs explained that:

*‘Sometimes it’s possible I could provide care without knowing* [the patient and family] *but I think it’s a real bonus — particularly when you’re making decisions about what to do.’* (MS, male GP in his 50s)

Intersubjective connection and relational knowledge is important in developing and practising clinical empathy, which is associated with improved patient outcomes.^37^ This requires practitioners to identify and understand the patient’s perspective and concerns and communicate and put it to therapeutic use. While research has shown it’s possible to generate clinical empathy in telephone consultations,^38^ the loss of non-verbal cues, disruption to the patient–GP relationship,^39^ and absence of the caring sensation of physical touch^40^ — the embodiment of caring^41^ — all influence the empathic connection. Evidence from psychotherapeutic literature suggests this results in a weaker therapeutic alliance^42^ and Hvidt has suggested this may occur due to the predominance of cognitive awareness in remote encounters over the embodied, perceptual awareness possible in person (Hvidt, 2024, personal correspondence).

Greenhalgh *et al* have highlighted how integration of different knowledge ‘types’ is vital in a multidisciplinary context to develop a coherent working knowledge that guides activity.^34^ However, some knowledge may only be available in person. One consultant described how it was impossible to *‘... walk around the* [virtual] *image we are shown ... ’* (NN, female consultant in her 50s) to see unwashed dishes or laundry that indicate how someone is really coping. Similarly they emphasised the importance of impromptu doorstep conversations with family members, which offer them a voice, provide additional information, and help build trust and confidence. These are impossible to conduct virtually. Inability to access such information and more superficial interpersonal relationships may reduce the richness of working knowledge generation, reducing the quality and coherency of care.

### Biomedical continuity: the illness episode

Biomedical tasks that were simple, transactional, and administrative were felt more amenable to digital or remote innovations than those that were complex, relational, or ‘human’. For example, monitoring devices and alarms or symptom trackers could support symptom control and one of the GPs described how during the COVID-19 pandemic a video call could give a rapid indication of a patient’s general comfort. Similarly, the specialty trainee described using virtual *‘eyeballing’* to detect if *‘something had changed’* (ST, female specialty trainee in her 30s) that might require further in-person assessment.

However, even ‘technical’ tasks lost a layer when conducted virtually. One of the specialty doctors highlighted how in person you could see, *‘... the pain in someone’s eyes as they sat down or walked into a room.’* (NN, female consultant in her 50s) and described how a hands-on assessment was essential to determine the most appropriate analgesia. Both patients and clinicians have a normative expectation of a clinical encounter^43^ and its fulfilment is associated with better outcomes and patient satisfaction.^44^ For many this includes a physical examination.^45,46^ Not only does touch aid with discernment and diagnosis of symptoms but ‘expressive touch’ improves communication.^47^ As embodied social beings that both experience the world and exist within it as objective beings,^48^ Field has identified how ‘touch hunger’ (evident in remote care) threatens our sense of ‘being-in-the-world’ and by extension our connectedness, growth, and flourishing.^40,49^ Thus, despite the logistical advantages of remote monitoring, these losses may outweigh the transactional benefits.

### Sociotechnical continuity: the distributed work of care

Enabling a coherent experience by joining up ‘work’ around the patient was felt to be a challenging but fundamental aspect of ‘good’ palliative care. Again participants described two levels: one transactional, technical, task-oriented and a deeper layer built on relational, intersubjective components. Overwhelmingly, participants emphasised the role technology could play by enabling the former while incompletely facilitating the latter.

Important distinctions were drawn between professional groups and their complementary roles. This highlights the importance of a multidisciplinary approach alongside the need to support the cultures and practices of individual professions as well as their understanding of other groups. Nurses highlighted communication, coordination, and logistical organisation around patients, while doctors also emphasised broader roles as educators, advocates, and leaders. In accordance with Starfield’s core elements of primary care — first contact, continuity of care, comprehensiveness, and coordination — one GP described having*:*

*‘... an overarching responsibility for the patient’s care and to be someone who has a holistic view to look at all aspects of things.’* (MS, male GP in his 50s)

Others described holding and contemplating complexity, balancing trade-offs, and taking responsibility for outcomes. One consultant reflected that this was enabled by:

*‘... something that medical training puts into your DNA so those things about asking questions, critical appraisal of the literature, doing some research, those things that move care on from what we’ve always done to thinking how we can do it better’*. (CV, female palliative consultant in her 50s)

Such distinctions reinforce MacIntyre’s depiction of a profession’s ‘internal goods’.^29^ Gabbay and le May have described how practitioners develop an understanding of these by drawing on the collective culture, knowledge, and activities within a community of practice to develop ‘mindlines’.^50^ Such knowledge has previously been enabled by spatiotemporal co-localisation, that is professionals working together in the same place at the same time. However, co-location and in-person multidisciplinary meetings have become infrequent and technology (email or electronic patient records) is often used to compensate. Giddens and Stones have highlighted how distanciation — the stretching of social systems over time and place^16^ — and disembedding — social activities occur at a distance^17^ — result in removal of the contextual specificities and immediacy and an increasing primacy for abstracted information.^16^ While logistically easier, such a move can result in relational and informational losses that reduce the efficacy and safety of sociotechnical continuity. One consultant highlighted that:

*‘... human beings are relational. Having a personal meeting with somebody moves things along so co-location in terms of doing things, being in the same building is the way to go.’* (NN, female consultant in her 50s)

Others reported that remote communication led to ‘Chinese whispering’ due to a loss of nuance and context when messages were left with ‘system administrators’. This caused incoherency and inefficiencies, duplication, frustration and distress for patients and professionals, alongside safety concerns, for example multiple medication prescriptions.

There was also a recognition of the need for an integrated system to *know* the patient. This could enable trust in the system itself and thus different professionals at particular points in time. One of the palliative care consultants reflected:

*‘The huge advantage of coordinated care is for the patients and their families. Also, for an integrated system it’s holding the story of who is this person. I was just finishing off some notes review with a student. I was saying to her, “some of these notes you’ll have some idea of who this person is who’s dying and some of them you’ll have none, and that doesn't mean that the staff didn't know the person”.’* (CX, female consultant in her 50s).

While participants reflected that electronic notes had improved information sharing, those in different organisations described a fantasy world where they could, *‘... see the whole patient story, history, holistic overview on one system.’* (CM team). They contrasted it with the reality of ‘... *poor and disjointed communication because of different IT systems ...’* (CM team) and the *‘... mixture of paper district nursing notes and electronic notes ...’* (CM team)*,* combined with challenges relating to the digital infrastructure such as inadequate network coverage. Additional effort was required to work around these limitations; for example, undertaking additional training to access different systems or using personal emails when the official software failed.^51^

Moreover there was an acknowledgement that while transactional, process information could be captured and transferred, there were limitations to the relational knowledge that could be exchanged. The speciality trainee highlighted:

‘*I can see the GP results and the referral letters they’ve written but I can’t see the consultations — I can’t see what’s been said.’* (ST, specialty trainee in her 30s)

This reflects the paucity of the digital facsimiles of an individual that can exist within an electronic patient record.^20^ Increasingly, participants described how this resulted in responsibility for care being handed back to the patient; for example, by empowering them with copies of their medical record through the NHS App or in written format — so-called *‘responsibilisation’*.^52^ However, there was also concern about the impact this exerted on patients and families at a difficult time.

### Ethical and/or political continuity: aligning with the community

One of the key challenges to effective integration relates to the dissemination of care across multiple communities, organisations, and professions with disparate values and aims. One of the consultants highlighted this, describing that:

*‘People might believe in integrated care on an abstract basis but being able and willing to deliver it requires generosity, requires trust, requires a sort of willingness to work together…cultural change and managers and leaders who are willing to provide the time and support for staff to do that.’* (CX, female consultant in her 50s)

The importance of leadership and clear lines of responsibility were highlighted by many interviewees.

Individuals are increasingly required to work together in interprofessional collaborations. Abbott and Hall have described how this requires them to open up and renegotiate the boundaries around their professional roles and practices,^31^ that is, engage in ‘boundary work’.^53,54^ Such boundaries are not existential, rather they emerge from interactions, supported by institutions, organisations and individuals.^31^. They may function either as barriers or enabling junctures^55^ with both intraprofessional and interprofessional relations determining the balance and thus extent of collaboration.^56^ While accommodating the activities of other roles or respecting their boundaries^57^ may create a ‘relational space’^58^ it may also generate rivalry, conflict, and reinforce a profession’s distinctions, particularly if they feel threatened.^59^

Similar challenges were reflected here, alongside efforts to combat them. One consultant described how good communication was rarely due to systems or processes but rather:

*‘... where there are strong interpersonal relationships between individuals and in teams that can overcome professional awkwardness.’* (WC, female consultant in her 30s)

Sometimes such relationships had developed naturally, facilitated by stable team structures. They resulted in a reciprocal trust that could also enable remote care. For example, care home staff described how:

*‘... because we have a good relationship with Dr X the video call visit wasn’t a problem because they trust us and we update them with information.’* (NM, female care home manager in her 50s.)

The GP also highlighted that:

*‘... trust and confidence in the person giving you the information* [enabled effective remote care home interactions] *— there are a couple of staff I trust implicitly.’* (MS, male GP in his 50s)

However, individuals also described taking a proactive, effortful approach to developing relationships and defining roles. For example the community nursing team used in-person meetings at different practices to raise awareness of their roles and ensure GPs were referring appropriately to them. All participants identified the additional work and effort required generally to combat the impacts of disembedding and distanciation and the additional toll this took on professionals.

## Discussion

### Summary

This qualitative exploration of the experiences of professionals involved in delivering integrated community palliative care has highlighted the challenges and complexities generated by remote and digital approaches and disseminated working practices. We have considered intersubjective, biomedical, sociotechnical, and sociopolitical factors that influence overall continuity and coherency of care. We have revealed a superficial transactional, task-based layer of care and a deeper, more relational component for which remote and digital approaches are less appropriate.

### Strengths and limitations

This study explores the views of professionals working within community palliative care. The semi-structured interviews combined with conversational prompts allowed for exploration of relevant themes while also highlighting emotive touchpoints for identification of salient issues. Ongoing discussions within the research team, participant member-checking, and subsequent discussion of the findings at a national academic healthcare services conference helped ensured rigor within the analytical process and interpretation of findings. However, all participants were purposively drawn from those caring for patients at a single Oxfordshire practice, although as previously mentioned, the teams operated at different geographical scales. It is likely that these findings do not directly represent the experience of other integrated palliative care teams, which may operate very differently and be influenced by a range of heterogenous contextual factors. Moreover, the majority of participants were female and tended to be more experienced professionals towards the mid-/later stages of their careers, which may also have resulted in presentation of particular viewpoints. Our aim was to give a rich picture of one integrated service and highlight the particular factors affecting the experiences of continuity in that setting. While these will necessarily be specific to the individual context, our use of the quadripartite framework helps consider how these particular experiences may be moved towards more general considerations for policymakers and service providers.

### Comparison with existing literature

In line with current UK policy, consultants echoed the need for integrated models to meet the increasing needs of our ageing population. However, like the wider chronic care literature, participants highlighted practical challenges to delivering such care.^18,19^ They emphasised technical limitations resulting from different commissioning decisions and variable digital infrastructures; for example, incompletely shared electronic records. Participants feared these exacerbated inequalities for vulnerable patients and introduced safety concerns. Such issues have been raised in relation to other aspects of remote working^21^ and pose burdens for patients and caregivers as additional effort is required to avoid harmful outcomes.^60^

Participants highlighted the complementarity of roles and importance of the multidisciplinary approach, which emphasises the need to support and respect contributions made by different professional groups. Interpersonal relationships were important in ensuring effective interprofessional integration. This generated a coherency that was seen as safer and gave patients and families a sense of security and care. The interprofessional relationships generated and transferred information that enabled relational and intersubjective knowledge of individuals; for example a tacit understanding about their guiding values and principles. While digital and remote approaches were thought sufficient for communicating transactional information, there was concern they could not fully replace these relational aspects. For example, in-person meetings were highlighted for the tacit, embedded, and ‘human’ forms of information they conveyed. Such relational awareness helped optimise the quality and holistic nature of the working knowledge generated around the patient’s management, enabling a rich integration and greater coherency.

This process of working knowledge generation is in line with recent work exploring other multidisciplinary teams^34^ and integrated palliative care systems, which emphasised the value of interprofessional networking (both vertically within teams and horizontally between teams) rather than standardisation of care. Along with interprofessional education, developing such knowledge enabled a sharing of norms and values about palliative care, developed interprofessional trust, and clarified leadership roles and responsibilities.^17^ Moreover, previous studies have highlighted the importance of this ‘epistemic dependence’ on different team members for optimal clinical decision making, particularly when it is co-constructed and disseminated between professionals.^22,23^

However, as in earlier studies, participants highlighted that effective integration is an effortful, time-consuming, and complex enterprise.^24^ It frequently requires articulation^25,26^ whereby tacit or embedded knowledge is used to bypass logistical or system barriers. Further system factors such as burdensome governance requirements presented additional challenges to these articulative efforts. Articulation around intersubjective knowledge and connections was perceived as particularly valuable. For example, despite the ease of remote meetings, one consultant deliberately arranged in-person discussions (with patients and other professionals) as they felt this enabled a deeper, relational communication that could not be transmitted effectively by virtual means, that is, it was lost through distanciation.

### Implications for future research and practice

Forming effective relationships and improving coherency within an increasingly strained healthcare system are challenging and will continue to be important areas for consideration by individual clinicians, service providers, and policymakers. As highlighted here, a lack of co-localisation means time is spent on remote communication and articulation activities with centralised administration ‘hubs’ that can impair the accuracy of messages. Participants here made a number of suggestions for how to improve this, which included more frequent in-person multidisciplinary meetings or shared remote networks (over MS Teams) as options to deepen connections. However, there was also a profound recognition of time constraints. More pragmatic suggestions focused around improved interorganisational sharing of electronic records, shared professional contact routes; for example, dedicated phone-lines and sharing of individual email addresses, and empowering patients to take a more formal role in mediating inter-professional communication. Although not directly raised here, the importance of modelling within communities of practice or as part of a training process has been demonstrated elsewhere.^27^ Therefore, ensuring exposure to successful interpersonal professional connections and integrated approaches may encourage their utilisation and aid a supportive ethos to promote their further uptake. Further work should continue to explore how best to enable these relational and sociotechnical activities that enable coherency within the context of challenging and evolving healthcare systems.

Here we have highlighted intersubjective, biomedical, sociotechnical, and sociopolitical factors that influence the provision of integrated, coherent palliative care. In particular we demonstrate the importance of promoting intersubjective connections and in-person meetings where possible to facilitate more relational domains of care alongside transactional. These also aid stronger interprofessional relationships to ensure generation of a high-quality multidisciplinary working knowledge. Remote and digital approaches may be appropriate for sharing more transactional information and knowledge but cannot fully replace the more relational aspects. High level policies may determine strategic aims, but resources are required to support the technologies, practices, and interprofessional interactions to enable integration on both a relational as well as transactional level.

## Data

Data may be available on request to the authors.
